# Supplementary material for: Identification of photoperiod-induced specific miRNAs in the adrenal glands of Sunite sheep (Ovis aries)
Source: Front Vet Sci. 2022 Jul 22;9:888207. doi: 10.3389/fvets.2022.888207 (PMC9354845; doi:10.3389/fvets.2022.888207)
Supplement: Supplementary file 1 [file Data_Sheet_1.ZIP › Supplementary file/miRDeep2 file.zip!/miRDeep2 file/Know_Structure/SP42A1/oar-mir-143.pdf]

miRBase precursor : oar-mir-143
Total read count : 222745
oar-miR-143 read count : 221135
remaining reads : 1610

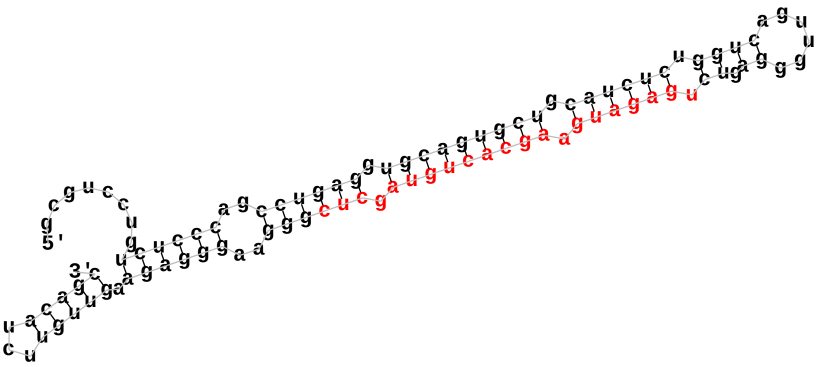

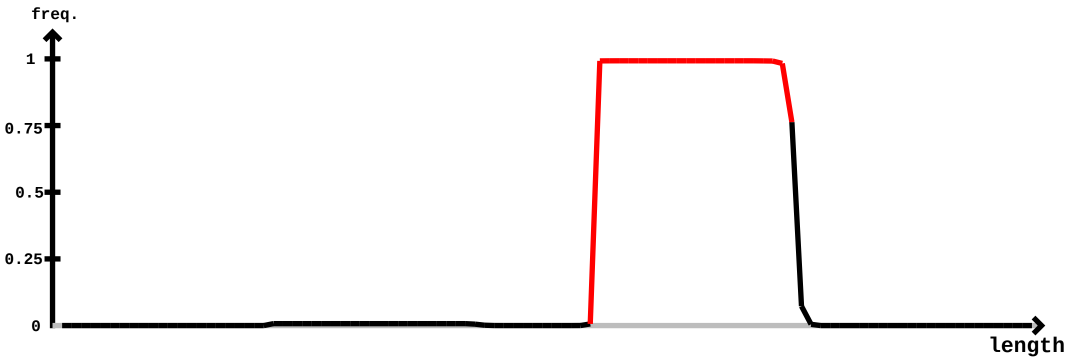


```
                                                      oar-miR-143
5'- gcguccugucucccagccugaggugcagugcugcaucucuggucaguugggagucugagaugaagcacuguagcucgggaagggagaaguuguucuacagc -3'  exp
    ........((((((..((((((.(((((((((.((((((.((((......)).)).)))))).))))))))).))))))..)))))).(((((...)))))   reads   mm   sample
    ....................aggugcagugcugcaucucugg...........................................................   4       0    seq
    ....................aggugcagugcugcaucucuggu..........................................................   1       0    seq
    .....................ggugcagugcugcaucucu.............................................................   2       0    seq
    .....................ggugcagugcugcaucucug............................................................   7       0    seq
    .....................ggugcagugcugcaucucugg...........................................................   421     0    seq
    .....................ggugcagugcugcaucucuggu..........................................................   883     0    seq
    .....................ggugcagugcugcaucucugguc.........................................................   262     0    seq
    .....................ggugcagugcugcaucucuggguca.......................................................   24      0    seq
    .....................ggugcagugcugcaucucuggucagu......................................................   1       0    seq
    .......................ugcagugcugcaucucuggucaguu.....................................................   1       0    seq
    ...........................................caguugggagucugagaugaagcacu................................   1       0    seq
    ...........................................caguugggagucugagaugaagcacuguagcuc.........................   1       0    seq
    ............................................aguugggagucugagaugaagcacu................................   1       0    seq
    ....................................................gucugagaugaagcacuguagcuc.........................   1       0    seq
    .....................................................ucugagaugaagcacuguagc...........................   1       0    seq
    .....................................................ucugagaugaagcacuguagcu..........................   7       0    seq
    .....................................................ucugagaugaagcacuguagcuc.........................   34      0    seq
    ......................................................cugagaugaagcacugu..............................   2       0    seq
    ......................................................cugagaugaagcacuguagc...........................   20      0    seq
    ......................................................cugagaugaagcacuguagcu..........................   301     0    seq
    ......................................................cugagaugaagcacuguagcuc.........................   972     0    seq
    ......................................................cugagaugaagcacuguagcucg........................   36      0    seq
    ......................................................cugagaugaagcacuguagcucgg.......................   1       0    seq
    .......................................................ugagaugaagcacugua.............................   77      0    seq
    .......................................................ugagGugaagcacugua.............................   1       1    seq
    .......................................................ugagaugaagcacuguG.............................   4       1    seq
    .......................................................ugagaugaagcacuUua.............................   2       1    seq
    .......................................................ugagaugaagUacugua.............................   1       1    seq
    .......................................................ugagaugaagcacuguU.............................   2       1    seq
    .......................................................ugagaugaagcacuguag............................   111     0    seq
    .......................................................ugagaugaagcacuguagc...........................   1968    0    seq
    .......................................................ugagaugaagcacuguagcu..........................   48783   0    seq
    .......................................................ugagaugaagcacuguagcuc.........................   152590  0    seq
    .......................................................ugagaugaagcacuguagcucg........................   15106   0    seq
    .......................................................ugagaugaagcacuguagcucgg.......................   1022    0    seq
```

oar-miR-143

gcguccugucucccagccugaggugcagugcugcaucucuggucaguugggagucugagaugaagcacuguagcucgggaagggagaaguuguucuacagc			
....ugagaugaagcacuguagcucggg......................	8	0	seq
....gagaugaagcacuguagc...........................	1	0	seq
....gagaugaagcacuguagcu..........................	17	0	seq
....gagaugaagcacuguagcuc.........................	39	0	seq
....gagaugaagcacuguagcucg........................	7	0	seq
....gagaugaagcacuguagcucgg.......................	1	0	seq
....agaugaagcacuguagcuc.........................	1	0	seq
....agaugaagcacuguagcucg........................	1	0	seq
....agaugaagcacuguagcucgg.......................	12	0	seq
....agaugaagcacuguagcucggg......................	1	0	seq
....gaugaagcacuguagcuc.........................	1	0	seq
....gaugaagcacuguagcucgg.......................	3	0	seq
....gaugaagcacuguagcucggga.....................	1	0	seq
....augaagcacuguagcucggga.....................	1	0	seq
